# Using support vector machine to explore the difference of function connection between deficit and non-deficit schizophrenia based on gray matter volume

**DOI:** 10.3389/fnins.2023.1132607

**Published:** 2023-03-27

**Authors:** Wenjing Zhu, Zan Wang, Miao Yu, Xiangrong Zhang, Zhijun Zhang

**Affiliations:** ^1^Department of Neurology, School of Medicine, Affiliated Zhongda Hospital, Research Institution of Neuropsychiatry, Southeast University, Nanjing, China; ^2^Affiliated Mental Health Center, Hangzhou Seventh People’s Hospital, Zhejiang University School of Medicine, Hangzhou, Zhejiang, China; ^3^Department of Geriatric Psychiatry, Affiliated Nanjing Brain Hospital, Nanjing Medical University, Nanjing, China

**Keywords:** deficit schizophrenia, functional connectivity, resting-state fMRI, gray matter volume, support vector machine

## Abstract

**Objective:**

Schizophrenia can be divided into deficient schizophrenia (DS) and non-deficient schizophrenia (NDS) according to the presence of primary and persistent negative symptoms. So far, there are few studies that have explored the differences in functional connectivity (FC) between the different subtypes based on the region of interest (ROI) from GMV (Gray matter volume), especially since the characteristics of brain networks are still unknown. This study aimed to investigate the alterations of functional connectivity between DS and NDS based on the ROI obtained by machine learning algorithms and differential GMV. Then, the relationships between the alterations and the clinical symptoms were analyzed. In addition, the thalamic functional connection imbalance in the two groups was further explored.

**Methods:**

A total of 16 DS, 31 NDS, and 38 health controls (HC) underwent resting-state fMRI scans, patient group will further be evaluated by clinical scales including the Brief Psychiatric Rating Scale (BPRS), the Scale for the Assessment of Negative Symptoms (SANS), and the Scale for the Assessment of Positive Symptoms (SAPS). Based on GMV image data, a support vector machine (SVM) is used to classify DS and NDS. Brain regions with high weight in the classification were used as seed points in whole-brain FC analysis and thalamic FC imbalance analysis. Finally, partial correlation analysis explored the relationships between altered FC and clinical scale in the two subtypes.

**Results:**

The relatively high classification accuracy is obtained based on the SVM. Compared to HC, the FC increased between the right inferior parietal lobule (IPL.R) bilateral thalamus, and lingual gyrus, and between the right inferior temporal gyrus (ITG.R) and the Salience Network (SN) in NDS. The FC between the right thalamus (THA.R) and Visual network (VN), between ITG.R and right superior occipital gyrus in the DS group was higher than that in HC. Furthermore, compared with NDS, the FC between the ITG.R and the left superior and middle frontal gyrus decreased in the DS group. The thalamic FC imbalance, which is characterized by frontotemporal-THA.R hypoconnectivity and sensory motor network (SMN)-THA.R hyperconnectivity was found in both subtypes. The FC value of THA.R and SMN was negatively correlated with the SANS score in the DS group but positively correlated with the SAPS score in the NDS group.

**Conclusion:**

Using an SVM classification method and based on an ROI from GMV, we highlighted the difference in functional connectivity between DS and NDS from the local to the brain network, which provides new information for exploring the neural physiopathology of the two subtypes of schizophrenic.

## 1. Introduction

Due to the absence of objective biological markers, schizophrenic diagnosis and treatment constitute one of the most complex clinical challenges of modern psychiatry, and extreme heterogeneity among patients further hinders the present research (Kirkpatrick et al., 2019; Goldsmith et al., 2021). Therefore, researchers try to parse the symptomatology of schizophrenia into more homogeneous diagnostic categories. Deficit schizophrenia (DS), proposed by Carpenter et al. (1988), is a homogeneous subtype characterized by a trait-like feature of primary and prominent negative symptoms. However, DS patients have more severe negative symptoms, worse long-term prognosis, greater cognitive impairment, lower recovery rates, and a high frequency of family history with schizophrenia (Kirkpatrick et al., 2001; Strauss et al., 2010; Potvin et al., 2021). Therefore, differentiating the two subtypes may have important implications for understanding the psychopathology and improving clinical interventions in these two subgroups of schizophrenia.

Magnetic resonance imaging (MRI) is widely used in a variety of mental illness and nervous system disease research, and provided early promise for the discovery of the neuroanatomical differences between the two subtypes of schizophrenia (Wang et al., 2015; Lei et al., 2019). Some studies found excessive DS-specific brain structural changes in gray matter volume (GMV), white matter volume, CSF volume, and cortical structure; including frontal, parietal, and temporal regions (Fischer et al., 2012; Podwalski et al., 2022). However, previous functional neuroimaging studies about the functional connectivity differences between these two subgroups of schizophrenia based on ROI from GMV are scarce.

Several studies have reported different patterns of FC abnormalities in DS and NDS patients, although the results remain inconclusive. For example, Ke et al. (2010) suggested that schizophrenia exhibiting positive symptoms had significantly increased leftward asymmetry of functional connectivity, but the negative symptom group exhibited increased rightward asymmetry of functional connectivity, and the strength of the asymmetry in these regions was correlated with symptom ratings. Zhou et al. (2019a) probed numerous abnormal FCs of nerve pathways between the two patient groups, mainly concentrated in the frontooccipital, frontotemporal, and insula-visual cortex, as well as the temporooccipital pathway. In addition, a recent study demonstrated abnormal patterns of FC in the nucleus accumbens network between DS and NDS (Zhou et al., 2021). Meanwhile, using the independent component analysis (ICA) method, further studies found the modular-level alterations in DS compared with the NDS and healthy controls, and the distinct and common disruptions mainly focus on SN, sensory motor network (SMN), DMN, and VN (Yu et al., 2017; Zhou et al., 2019b; Fan et al., 2022). Nevertheless, the limitations of these earlier studies about FC of DS/NDS were that such functional brain connectivity results can be biased by the selection of seed region or spatial network template. Alternatively, there has been a paucity of studies mentioning the thalamocortical imbalance in the two subtypes of schizophrenia which is characterized by prefrontal-thalamic hypoconnectivity and sensorimotor-thalamic hyperconnectivity observed in resting-state fMRI studies, and has been implicated in the pathophysiology of schizophrenia (Anticevic et al., 2015; Avram et al., 2018; Wu et al., 2022).

Support vector machine (SVM), which is one of the most commonly used machine learning (ML) methods in pattern recognition (Lei et al., 2020), has been widely utilized as a powerful computational approach to classify schizophrenic patients from healthy controls and predict outcomes based on neuroimaging data (Wang et al., 2018; Chang et al., 2021). There are also very few studies that use SVM to classify and predict DS and NDS. Based on tryptophan catabolites and Consortium To Establish a Registry for Alzheimer’s disease features, Kanchanatawan et al. (2018) used SVM to strongly segregate deficit from non-deficit schizophrenia and healthy controls. On the other hand, 144 patients were successfully classified as deficit patients using an SVM classifier based on severity, persistence over time, and possible secondary sources (e.g., depression) of negative symptoms in the research of Fervaha et al. (2016). However, few studies use magnetic resonance data to classify DS and NDS. Based on effective feature selection of these image data, SVM can find more objective seed regions for functional connectivity analysis of schizophrenia. In the present study, we applied SVM to discriminate DS from NDS using their GMV data. Then, the high-weight classified brain regions were used as seed points in whole-brain FC analysis and thalamic FC imbalance analysis of DS and NDS. Finally, we investigated the relationship between altered FC and clinical scale. We hypothesized that (1) Using the SVM classification method, this study can achieve the classification of two subtypes of schizophrenia and obtain key brain regions from analysis of GMV. (2) This study can find the difference in functional connectivity between DS and NDS from the local to the brain network, which provides new information for exploring the neural physiopathology of the two subtypes of schizophrenic. (3) Observed altered FC between DS, NDS, and HC will also be correlated with clinical scale.

## 2. Materials and methods

### 2.1. Participants

A total of 86 naturally right-handed Han Chinese participants ranging in age from 20 to 65 years were recruited in this study, 48 schizophrenia patients and 38 matched healthy controls. All the patients were recruited from the psychiatric rehabilitation unit of Yangzhou Wutaishan Hospital, Jiangsu Province, China. The inclusion criteria for the patients are: (1) An explicit diagnosis of schizophrenia according to the Diagnostic and Statistical Manual of Mental Disorders, Fifth Edition (DSM-V); (2) Presenting stable psychiatric symptoms after antipsychotic medication for at least 12 months before participation. The exclusion criteria for the patients are: (1) Severe neuropsychiatric comorbidities, such as head trauma or intellectual disability; (2) Alcoholism or substance abuse; (3) Physical therapy including electroconvulsive therapy; (4) Contraindications for MRI; (5) Noticeable head motion (>3 mm in translation or 3^°^ in rotation). One patient was excluded because of large head motion, the remaining 47 patients were enrolled in the final research. According to the Chinese version of the Schedule for Deficit Syndrome (SDS) (Wang et al., 2008), patients were divided into two groups: DS and NDS groups, including 16 DS patients and 32 NDS patients, respectively. Patients with two of the following symptoms present at a moderately severe level and persistent over 12 months were defined as having DS: restricted affect, diminished emotional range, poverty of speech, curbing of interests, diminished sense of purpose, and diminished social drive; all the symptoms required the absence of secondary sources (e.g., medication side effects, depression, etc.). In total, 38 gender-, age-, and handedness-matched HC volunteers were recruited from the communlocal advertisements. Unstructured clinical interviews were conducted to exclude HCs who had a history of organic brain disorders, intellectual disability, or severe head trauma as well as a history of personal or family psychiatric disorder. All participants gave informed consent to participate in this study, which was approved by the Institutional Ethical Committee for clinical research of Zhongda Hospital Affiliated with Southeast University.

### 2.2. Assessments of clinical symptoms and antipsychotic treatment

The severity of the schizophrenic symptoms was evaluated by the Brief Psychiatric Rating Scale (BPRS), the Scale for the Assessment of Negative Symptoms (SANS), and the Scale for the Assessment of Positive Symptoms (SAPS). All patients received antipsychotic medications according to the case clinician’s preference for at least 12 months before participation. The details of treatment were assessed from patients’ or their guardians’ reports and hospital records. The dosage of antipsychotic medication of each patient was recorded and converted to chlorpromazine-equivalent mean daily dosages (MDD) (Woods, 2003). Table 1 illustrates the clinical and demographic data of all participants.

**TABLE 1:** Demographic and clinical characteristics.

	DS (*n* = 16)	NDS (*n* = 31)	HC (*n* = 38)	^b^T/F/χ^2^	*P*-value
Gender (Male/female)^a^	9/7	16/15	20/18	0.09	0.954
Age	50.63 ± 9.73	45.13 ± 5.33	45.71 ± 9.63	2.54	0.085
Educational years	8.75 ± 2.05	9.29 ± 1.95	10.74 ± 2.73	5.34	0.007
BPRS-T	32 ± 2.81	27.32 ± 2.15	–	6.36	0.000
SAPS-T	9.06 ± 3.68	10.13 ± 4.51	–	–0.82	0.419
SANS-T	55.25 ± 8.06	32.16 ± 5.71	–	11.39	0.000
Antipsychotic (*n*)^c^	16	31	–	–	–
Antipsychotic medication^d^, day/mg	446.23 ± 259.21	483.82 ± 238.03	–	0.86	0.591
FD value	0.13 ± 0.11	0.12 ± 0.17	0.11 ± 0.24	2.57	0.286

DS, deficit schizophrenia; NDS, non-deficit schizophrenia; HC, healthy control; BPRS, Brief Psychiatric Rating Scale; SAPS, Scale for the Assessment of Positive Symptoms; SANS, Scale for the Assessment of Negative Symptoms; FD, frame-wise displacement, used to evaluate head motion during scanning.

^a^Data are presented as mean ± standard deviation except gender.

^b^Comparisons were performed with a chi-square test for the variable of gender and independent samples t-tests for other variables. Adjusted age and education were employed as covariates.

Bonferroni correction was used for post-hoc comparisons.

^c^All participants were taking atypical antipsychotics.

^d^Chlorpromazine equivalent doses were calculated.

### 2.3. Multimodal MRI data acquisition

All participants were scanned by a 3T MR system (GE HDx, Chicago, IL, USA) with an eight-channel phased array head coil in the Subei Hospital of Jiangsu Province, Yangzhou, China. T1-weighted images were acquired by three-dimensional spoiled gradient echo sequence as follows: repetition time (TR) = 11.94 ms, echo time (TE) = 5.044 ms, flip angle = 15^°^, slice thickness = 1 mm without gap, number of slices = 172, field of view (FOV) = 240 × 240 mm, matrix size = 256 × 256. In addition, R-fMRI data were acquired with a gradient recalled echo echo-planar imaging (GRE-EPI) sequence: repetition time (TR) = 2,000 ms, echo time (TE) = 25 ms, flip angle = 90^°^, number of slices = 35, field of view (FOV) = 240 × 240 mm, slice thickness = 4 mm without gap, matrix size = 64 × 64, voxel size = 4 × 4 × 4 mm^3^, 240 volumes. All participants were asked to lie quietly awake in the scanner with their eyes closed, and their heads were cozily positioned with cushions inside the coil during the MRI scan to minimize head motion.

### 2.4. Image preprocessing and VBM analysis

The Statistical Parametric Mapping 8 (SPM8)^1^ and Data Processing & Analysis for Brain Imaging (DPABI)^2^ were applied to preprocess the fMRI data in MATLAB.^3^ The data preprocessing includes the following steps: (1) discarding of the first 10 volumes to achieve equilibrium and a steady-state; (2) slice timing correction; (3) realignment: head motion parameters were computed by estimating the translation in each direction and the angular rotation on each axis for each volume, we required the translational or rotational motion parameters less than 3 mm or 3^°^, The frame-wise displacement (FD), which indexes the volume-to-volume changes in head position was also calculated; (4) spatially normalized: individual structural images were firstly co-registered with the mean functional image; then the transformed structural images were segmented and normalized to the Montreal Neurological Institute (MNI) space using a high-level non-linear warping algorithm which use the exponentiated Lie algebra (DARTEL) technique (Ashburner, 2007) to acquire the diffeomorphic anatomical registration, Finally, each functional volume was spatially normalized to MNI space using the deformation parameters estimated during the above step and resampled into a 3 mm cubic voxel; (5) Nuisance covariates regression: six head motion parameters, cerebrospinal fluid signals, white matter signals, and global mean signals were regressed from the data as corrected values; (6) spatial smoothing with a Gaussian kernel of 8 × 8 × 8 mm^3^.

T1-weighted structural brain images were visually inspected for motion and artifacts before VBM analysis and for segmentation errors prior to inclusion in the group analyses. Firstly, the VBM8 toolbox^4^ was adopted to preprocess and segment images that passed quality control into gray matter, white matter, and cerebrospinal fluid. Then, the images underwent non-linear normalization to MNI space with the DARTEL algorithm after bias correction and segmentation. The images were modulated by non-linear warping only. Finally, the normalized gray matter images were smoothed with a 6mm kernel and used as characteristic parameters for the SVM method to determine the ROIs for functional connectivity.

### 2.5. Determination of ROI with SVM method and functional connectivity analysis

In order to classify the three groups of HCs, DS, and NDS, and to find the brain regions with GMV differences between the two subtypes of schizophrenia through imaging data, we adopted the Pattern Recognition for Neuroimaging Toolbox (PRoNTo)^5^ (Schrouff et al., 2013) which we ran on Matlab, aiming to facilitate the interaction between machine learning and neuroimaging communities. Based on PRoNTo software, we then applied linear kernel Support vector machines (SVM) which is one of the most commonly used machine learning techniques in neuroimaging to achieve the classification. In the SVM training phase, Spatiotemporal images of the GMV for each subject from two subtypes are calculated as features, and weights are assigned to these features for maximal separation between the groups using a hyperplane, which serves as the decision boundary. Classification labels are determined by the sign of the total feature weights multiplied by the test sample. We use the default soft-margin parameter of C = 1. Meanwhile, a 10-fold cross-validation scheme we employed to assess the performance of models generated by these algorithms. Subsequently, we calculated the brain regions that accounted for the top 1% of classification weights, and the numerical values obtained from these high classification weight GMV regions were extracted with the Resting-State fMRI Data Analysis Toolkit (REST),^6^ using these brain regions with high classification weight as masks. Thereafter, GMV values were converted to Z-values *via* Fisher Z transformation and used for the correlation analysis. The study then selected the brain regions whose GMV values negatively correlated with the SANS score as seed points to construct the FC analysis.

In the present study, these high-weight classified brain regions between DS and NDS were used as regions of interest for whole-brain-based FC analysis. Pearson correlation analysis was carried out to obtain the correlation coefficient (r-value) of the mean time series between the seed point and the whole-brain voxel in each participant. Fisher Z transformation was then performed to convert the r value to the Z value, which conforms to the normal distribution. Part of the numerical values resulting from regions of altered FC was extracted with REST using altered regions as masks and were later used for correlation analysis.

### 2.6. Statistical analyses

The statistical descriptive analyses of demographic and clinical scales were conducted using the SPSS 17.0 software package. These parameters were compared with the chi-squared test, the two-sample *t*-tests, and the analysis of variance (ANOVA) appropriately. ANOVA was performed using the DPABI to investigate differences in whole-brain FC among DS, NDS, and HC groups. Age and education were used as covariates. False discovery rate (FDR) correction was performed for multiple comparisons at the voxel level. The statistical threshold was set at a corrected *p* < 0.05. The associations between the valuable GMV and FC of brain regions and Clinical scale value in both the DS and NDS groups were performed with Partial correlation analysis (age, education, and chlorpromazine equivalent as covariates).

## 3. Results

### 3.1. Demographic and clinical characteristics

Table 1 describes the demographic, clinical scale, and head motion data of 16 DS patients, 31 NDS patients, and 38 HCs. There was no significant difference between the three groups in terms of age, gender, and head motion. Compared with the DS patients, patients with NDS presented higher scores in SAPS-T and lower scores in BPRS-T and SANS-T. Both groups of patients received antipsychotic medications including olanzapine, risperidone, quetiapine, clozapine, and other commonly used antipsychotics, and there was no statistical difference in the chlorpromazine equivalent doses of the two groups (*p* > 0.05).

### 3.2. Classification performance and regions contributing to discrimination between DS and NDS

**FIGURE 1:**
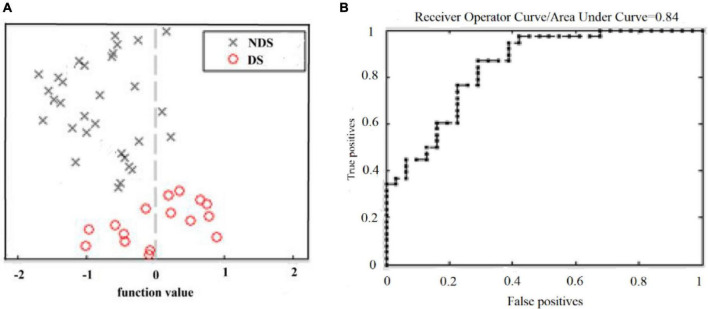
Classification between DS and NDS based on GMV. **(A)** Classification plot for training on DS/NDS data and testing on DS/NDS data using GMV. **(B)** ROC curve obtained by classifying DS and NDS using an SVM classifier. DS, deficit schizophrenia; NDS, non-deficit schizophrenia; GMV, gray matter volume; ROC, receiver operating characteristic curve; SVM, support vector machine.

**FIGURE 2:**
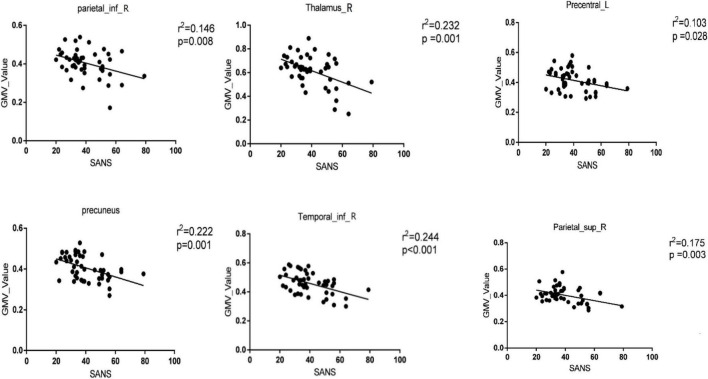
Significant correlations between the gray matter volume (GMV) values of brain regions with classification weights in the top 1% and clinical features in the whole patient group [deficit schizophrenia (DS) group and non-deficit schizophrenia (NDS) group]. The significance threshold was set at *p* < 0.05 (uncorrected). Parietal_inf_R, right inferior parietal lobule; Thalamus_R, right thalamus; Precentral_L, left precentral gyrus; Temporal_inf_R, right inferior temporal gyrus; Parietal_sup_R, right upper parietal lobule.

The classification performance for the GMV feature is summarized in Table 2. Total classification accuracy between DS and NDS was 78.6%, and the illustration of classification is presented in Figure 1, ROC curve of GMV was also obtained with the SVM classifier [Figure 1B, Area Under Curve (AUC) = 0.84]. Regions that contributed to GMV discrimination and accounted for the top 1% of classification weights included right inferior parietal lobule, right upper parietal, left upper parietal, left precentral gyrus, right precentral gyrus, postcentral gyrus, paracentral lobule, precuneus, right inferior temporal gyrus, right thalamus, and cerebellum (Supplementary Figure 1). Then, the GMV values of these 11 high-weight classified brain regions were extracted with REST and were later used for correlation analysis. The results of correlation analysis suggest that six brain regions whose GMV values were negatively correlated with the SANS scale included the right inferior parietal lobule, right upper parietal lobule, left precentral gyrus, precuneus, right inferior temporal gyrus, and right thalamus (shown in Figure 2). But the GMV values were not significantly correlated with other scales.

**TABLE 2:** Classification results of deficit schizophrenia (DS)/non-deficit schizophrenia (NDS)/healthy control (HC) using support vector machine (SVM) classifier based on gray matter volume (GMV).

Groups	Total accuracy	Balanced accuracy (BA)	BA *p*-value	AUC
DS/NDS	78.60%	72.63%	0.003	0.84
DS/HC	84.63%	76.05%	0.001	0.85
HC/NDS	83.26%	75.59%	0.001	0.84

AUC, area under curve.

### 3.3. Functional connectivity

**FIGURE 3:**
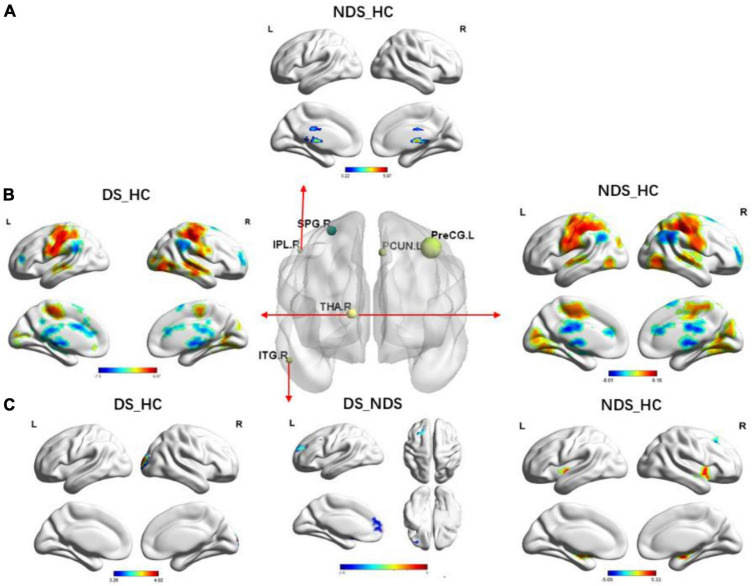
Group comparisons of functional connectivity (FC) in six region of interests (ROIs) among deficit schizophrenia (DS), non-deficient schizophrenia (NDS), and health controls (HC). **(A)** Group analyses of the FC in the right inferior parietal lobule between NDS and HC groups. The significance threshold was set at *p* < 0.05 after false discovery rate (FDR) was corrected (voxel *p* < 0.05, cluster size ≥ 146). **(B)** Group analyses of the FC in the right thalamus between patients and HC groups. The significance threshold was set at *p* ≤ 0.05 after FDR was corrected (voxel *p* < 0.05, cluster size ≥ 200). **(C)** Group analyses of the FC in the right inferior temporal gyrus between patients and HC groups. The significance threshold was set at *p* < 0.05 after FDR was corrected (voxel *p* < 0.05, cluster size ≥ 162/100/100). The blue regions indicate the regions where patients had lower FC compared to HC; the red areas show the regions where patients had greater FC compared to HC, when DS vs. NDS, and the blue regions indicate the regions where DS had lower FC compared to NDS. IPL.R, right inferior parietal lobule; THA.R, right thalamus; ITG.R, right inferior temporal gyrus; FC, functional connectivity.

**FIGURE 4:**
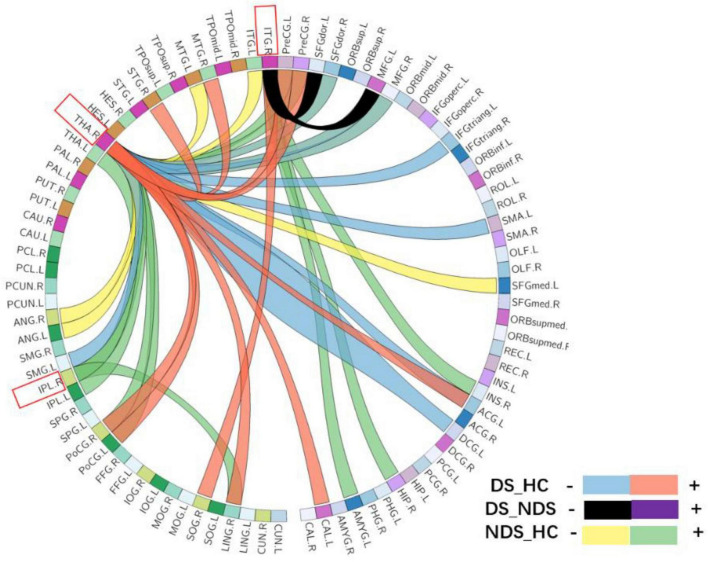
The circular graph obtained by comparing the functional connections between the three groups based on the three region of interests (ROIs) (IPL.R, THA.R, ITG.R). IPL.R, right inferior parietal lobule; THA.R, right thalamus; ITG.R, right inferior temporal gyrus.

Functional connectivity analysis based on three seed points between patients and HC. As shown in Figures 3, 4, some abnormal FCs were found among the three groups. Compared to HC, the FC of IPL.R with bilateral thalamus and lingual gyrus, of ITG.R with SN was enhanced in NDS. Both DS groups and NDS groups were found to have an imbalance in thalamic FC which was enhanced between THA.R and SMN but decreased between THA.R and the frontotemporal area. In addition, the FC enhanced between the THA.R and VN, between ITG.R and right superior occipital gyrus in DS group. The FC decreased between the ITG.R and the left superior and middle frontal gyrus in the DS group when compared with NDS.

### 3.4. Altered FC of THA-R between DS, NDS, and HCs, and its relationship with clinical symptoms

**FIGURE 5:**
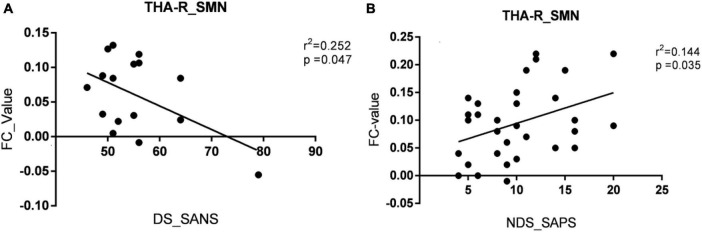
The relationships between altered functional connectivity (FC) value of right thalamus (THA-R) with clinical symptoms. **(A)** The relationships between the FC value of THA.R and SMN with the Scale for the Assessment of Negative Symptoms (SANS) score in the deficient schizophrenia (DS) group. **(B)** The relationships between the FC value of THA.R and SMN with the Scale for the Assessment of Positive Sympt (SAPS) score in the non-deficient schizophrenia (NDS) group. The significance threshold was set at *p* < 0.05. SMN, sensory motor network. THA-R_SMN, FC of right thalamus and the sensory-motor network; DS_SANS, scoring on the SANS in the DS group; NDS_SAPS, scoring on the SAPS in the NDS group.

The brain regions with altered FC of THA-R were mentioned in Table 3 and the relationship was mentioned in Figure 5, results show that compared with HC, the FC enhanced between THA.R and SMN, but decreased between THA.R and frontotemporal area both in the DS and NDS groups. In addition, the FC between the THA.R and VN was also enhanced in the DS group. The FC value of THA.R and SMN in the DS group was negatively correlated with the SANS score (*P* < 0.05). But the FC value of THA.R and SMN in the NDS group was positively correlated with the SAPS score (*P* < 0.05).

**TABLE 3:** Altered regions of functional connectivity (FC) analysis based on the region of interest (ROI) of right thalamus (THA-R).

Brain regions	Peak MNI coordinate			F/t	Cluster size
	X	Y	Z		
**ANCOVA**					
L-postcentral/L-precentral/L-parietal_inf	–51	–21	36	53.13	1,158
R-postcentral/R-precentral	45	–30	60	43.39	943
R-temporal_mid/R-temporal_sup	57	–15	–6	27.23	171
R-occipital_inf/R-occipital_mid	42	–78	–3	27.54	101
R-lingual	18	–45	–12	31.69	250
L-cingulum_ant	0	15	27	27.54	84
**DS vs. HC**					
L-postcentral/L-precentral/L-parietal_inf/ L-paracentral lobule	-51	–21	36	6.68	1,319
R-postcentral/R-precentral	45	–27	60	6.87	1,039
R-lingual/R-calcarine	18	–75	–12	5.20	625
R-temporal_mid/R-temporal_sup	57	–33	3	5.41	246
L-cingulum_ant	0	15	27	–5.07	142
**NDS vs. HC**					
B-postcentral/B-precentral/L-parietal_inf	–48	–24	45	9.16	6,210
B-lingual	45	–75	0	6.34	1,976
L-occipital_inf	–48	–72	–6	5.28	102
R-frontal_sup/R-frontal_mid	30	57	18	–4.51	95
B-cingulum_ant	6	30	18	–4.62	173
B-augular	57	–48	36	–5.78	441
DS vs. NDS	None	–	–	–	–

## 4. Discussion

The main findings of the current work are as follows: (1) The classification accuracy of SVM was 78.60% between DS and NDS, and 84.63% between DS and HC, six important regions of interest related to clinical symptoms were obtained from the classification between DS and NDS; (2) Compared to HC, the FC increased between IPL.R and bilateral thalamus, and lingual gyrus, between ITG.R and SN in NDS. Alternatively, the FC enhanced between the THA.R and VN, ITG.R, and right superior occipital gyrus in the DS group. Compared with NDS, the FC between the ITG.R and the left superior and middle frontal gyrus decreased in the DS group; (3) Thalamic FC imbalance analysis suggested that the FC enhanced between THA.R and SMN, but decreased between THA.R and frontotemporal area both in DS and NDS groups. Interestingly, the FC value of THA.R and SMN in the DS group was negatively correlated with the SANS score, but the FC value of THA.R and SMN in the NDS group was positively correlated with the SAPS score. To the best of our knowledge, this is the first study using machine learning methods to classify the two subtypes of schizophrenia from normal controls based on GMV and perform whole-brain functional connectivity analysis based on ROI with less bias obtained from the classification. This study further explores the neural physiopathology of the two subtypes of schizophrenia.

There are few previous studies based on electrophysiological, neurocognitive testing, oxidative stress toxicity, neuroimmune and other parameters, using machine learning methods to classify DS, NDS, and normal controls; correspondingly, the classification accuracy is in the range of 70–85% (Kanchanatawan et al., 2018; Maes et al., 2020; Taylor et al., 2020). Unlike these past studies, the current study extracts features from GMV image maps, uses the SVM method to classify DS and NDS, the six important brain regions for distinguishing DS and NDS, and their GMV values were negatively correlated with the SANS scale, including right inferior parietal lobule, right upper parietal, left precentral gyrus, precuneus, right inferior temporal gyrus, and right thalamus.

The results of previous MRI studies of DS and NDS have shown that the frontal lobe, temporal lobe, and precuneus are the main areas of gray matter reduction, the degree of reduction was also negatively correlated with negative symptoms, results of this research are consistent with these findings. Positive symptoms such as hallucinations, delusions, and thinking disorders are important differences between DS and NDS. Meanwhile, the temporal lobe is related to auditory and language processing and thought, so it may be the material basis for the difference in positive symptoms between the two types of patients. The precuneus plays a major role in higher-order self-processes and the attribution of emotion to self and others. The precuneus gray matter volume is significantly different between DS and NDS subtypes, which may explain why patients with deficient schizophrenia have difficulty in emotional expression.

Several functional connectivity and local network abnormalities, involving the thalamus, inferior parietal gyrus, salience network, sensory motor network, and visual network, were found in both the DS and NDS groups compared with the HCs. It has been reported previously that most of these regions have abnormal functional connectivity in patients with schizophrenia (Skudlarski et al., 2010; Zalesky et al., 2011). The thalamus is an important nerve nucleus within the brain and is a secondary conduction pathway that plays the role of upward conduction for all sensations except smell. In addition, the thalamus is also involved in people’s emotional activities, thalamus damage will lead to emotional disorders, but also cognitive function, speech function decline, and so on (Avram et al., 2018; Wu et al., 2022). In the present study, FC between the THA.R and VN was enhanced in DS group when compared with HC, which represents an abnormal visual process in order to improve neurocognitive function in DS patients, thus may indicate a unique neuropathological mechanism in this schizophrenia subgroup. The present findings also demonstrated hypo-connectivity between the right ITG and left superior/middle frontal gyrus in the DS group relative to the NDS group. Previously published studies have shown that the right ITG, important for language formulation and face perception (Schultz et al., 2000; Dien et al., 2013), has been reported to have volume reduction in DS patients. Furthermore, abnormally increased activation of the right ITG was found to be related to deficits in facial recognition and interpersonal communication in autistic patients (Schultz et al., 2000), which are phenotypically similar to the negative symptoms of schizophrenia. Part of the frontoparietal circuitry comprises the mirror neuron system, which is activated during basic emotion understanding and emotion experience sharing. Therefore, all these combined factors can be analyzed to show that the hypo-connectivity between the right ITG and frontal-parietal circuit in DS patients is likely to be a potential neural mechanism for the prominence of negative symptoms.

Published data (Anticevic et al., 2015; Li et al., 2017; Avram et al., 2018) support the hypothesis that thalamocortical imbalance may be one inherent feature of schizophrenia. The results of this study are consistent with the hypothesis that the FC enhanced between THA.R and SMN, but decreased between THA.R and frontotemporal area both in DS and NDS groups when compared with HC. In addition, the FC value of THA.R and SMN in the DS group was negatively correlated with the SANS score, but the FC value of THA.R and SMN in the NDS group was positively correlated with the SAPS score. Previous studies have shown that the SMN mainly regulating sensory and motor functions, is the core network that is vulnerable to dysfunction in emotional functions, emotion recognition, and cognitive functions of psychiatric disorders (Wood et al., 2016; Davis et al., 2017). The conclusions have also been presented in previous studies, for instance, Cheng et al. (2015) reported an association between sensorimotor cortico-thalamic hyperconnectivity and negative symptoms. Anticevic et al. (2014) reported an association with general psychopathology. Combining these studies, it can be speculated that the presence of persistently progressive negative symptoms in patients with DS further exacerbates sensorimotor cortico-thalamic hyperconnectivity.

In conclusion, the present study investigates functional connectivity alterations between DS and NDS from the local to the whole and their relationships with clinical symptoms based on the regions of interest obtained by the SVM classifier, and the results obtained a relatively high classification accuracy. Based on the ROIs, including IPL.R, ITG.R, and THA.R, this study demonstrated the FC between the THA.R and VN enhanced in the DS group when compared with HC, FC between the right ITG and left superior/middle frontal gyrus decreased in DS group relative to NDS group. The findings of this study corroborate the previous conclusion of the hypothesis that thalamocortical imbalance in both of the two subtypes of schizophrenia. In addition, the FC value of THA.R and SMN in the DS group was negatively correlated with the SANS score, but the FC value of THA.R and SMN in the NDS group was positively correlated with the SAPS score, which deepens the understanding of the pathological mechanism of the two subtypes of schizophrenia.

## 5. Limitations

Some limitations of this study should be addressed. First, our study enrolled a small sample size. Larger samples in the future are needed to confirm current findings. Second, only one machine learning method of support vector machine is used for classification in this study, and the classification accuracy is not very high. So later research can improve the performance of the classifier. Third, this study only included the right thalamus in the study of thalamocortical imbalance, which will make the attribution of thalamic FC imbalance analysis in the two subtypes of schizophrenia incomplete.

## Data availability statement

The original contributions presented in this study are included in the article/Supplementary material, further inquiries can be directed to the corresponding authors.

## Ethics statement

The studies involving human participants were reviewed and approved by the Institutional Ethical Committee for Clinical Research of Zhongda Hospital Affiliated to Southeast University. The patients/participants provided their written informed consent to participate in this study.

## Author contributions

ZZ and XZ supervised the present study. WZ and ZW performed the analysis and wrote the manuscript. XZ and MY helped to collect data. All authors contributed to the article and approved the submitted version.

## Footnotes

1
http://www.fil.ion.ucl.ac.uk/spm/software/spm8/

2
http://rfmri.org/dpabi

3
http://www.mathworks.com/products/matlab/

4
http://dbm.neuro.uni-jena.de/vbm

5
www.mlnl.cs.ucl.ac.uk/pronto

6
http://resting-fmri.sourceforge.net/
