# Supplementary material for: Using support vector machine to explore the difference of function connection between deficit and non-deficit schizophrenia based on gray matter volume
Source: Front Neurosci. 2023 Mar 27;17:1132607. doi: 10.3389/fnins.2023.1132607 (PMC10083255; doi:10.3389/fnins.2023.1132607)
Supplement: Supplementary file 1 [file Data_Sheet_1.docx]

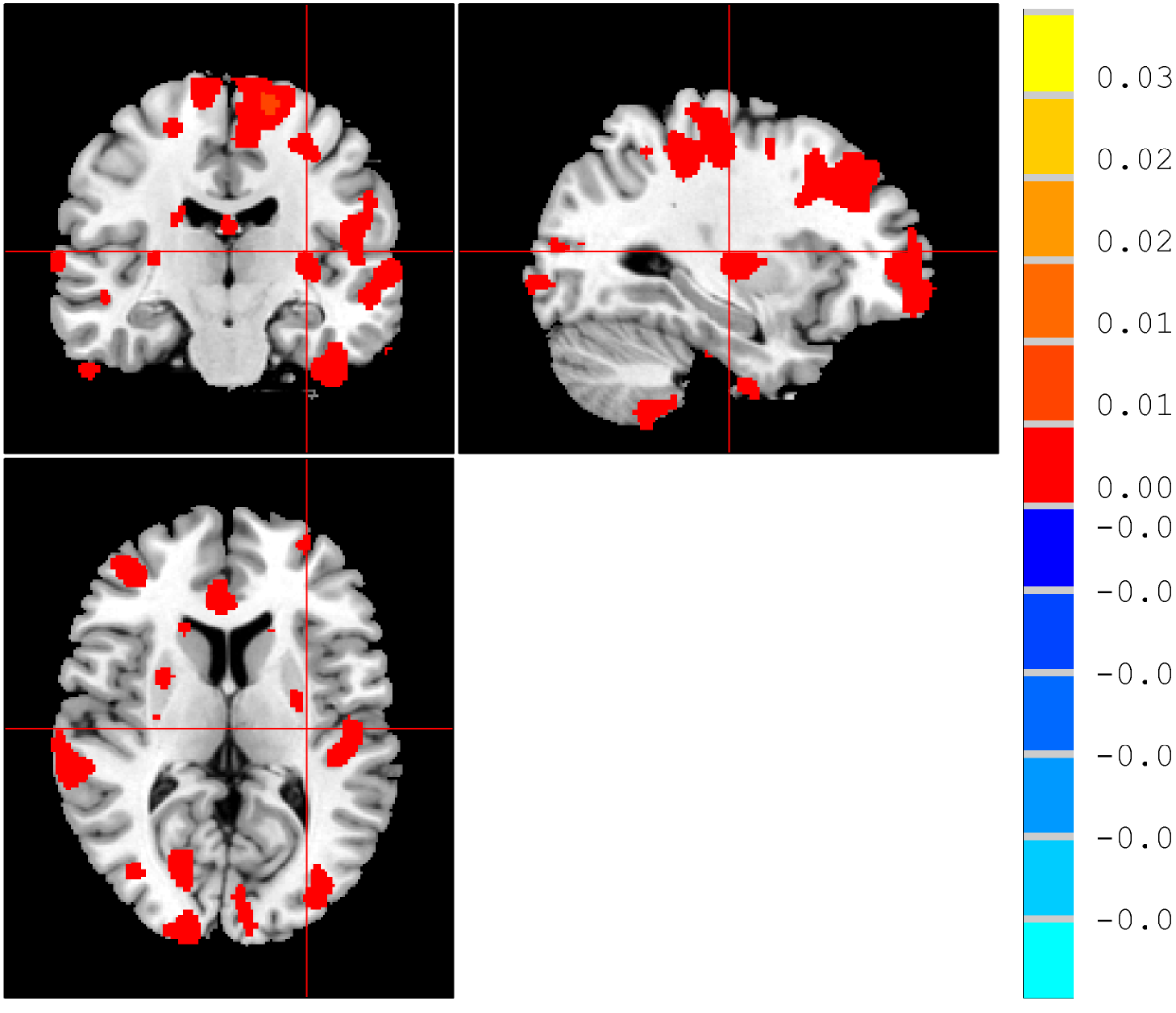


Fig.6 Brain area distribution map with weight accounting for the first 1% in DS and NDS classification (three-dimensional brain map).
